# Creosote in Phthisis Pulmonalis

**Published:** 1899-04

**Authors:** L. H. Warner

**Affiliations:** Brooklyn, N. Y.


### Selections.

### CREOSOTE IN PHTHISIS PULMONALIS.

By L. H. WARNER, M. D., Brooklyn, N. Y.

(*From the Denver Medical Times.*)

AFTER a brief review of creosote and guaiacol and the various methods of employing these products, the author says :

In the treatment of phthisis the administration of creosote causes the fever and cough to diminish and the patient to improve in appetite and flesh. On examination of the pulse it will be noted there is a smallness and rapidity indicating an increased anemia produced by the powerful action of creosote. When creosote alone is used life is made more comfortable to the patient, but it causes an earlier termination. If in combination with tonics less anemia is produced. It has antifermentative powers and although it may not kill bacteria, it destroys their ptomaines and renders their action nontoxic and inert. In the stomach of the consumptives a pathological fermentation is at all times going on, and this process is overcome by the action of creosote. It takes oxygen from the blood, and is changed into carbolates and oxalates, as a result of oxydation, thus causing the blood to assume a deeper color. In the treatment of phthisis it becomes of especial value if reinforced by nuclein. Nuclein increases the number of white blood corpuscles and is therefore a valuable agent in combating tuberculosis in its initial stage. Reviewing the aforementioned facts, we have creosote, guaiacol, nuclein and tonics as factors in the treatment of phthisis pulmonalis. How and in what proportion can they be best combined to become efficient in the treatment of this disease? Beef, milk and wheat peptonised, with creosote and guaiacol, otherwise known as liquid peptonoids with creosote, is an eligible method of administering the above in combination. Each tablespoonful contains two minims of pure beechwood creosote and one minim of guaiacol combined with the nutrient and reconstituent properties of liquid peptonoids. In two different hospitals the entire consumptive wards were placed on this remedy with most excellent results and it will be necessary to quote but a few of the many cases under observation:

CASE I.—M. P., female, aged 49, admitted to hospital June 2, 1898; family history, tubercular. For some years patient has been troubled with severe attacks of cough, resulting from an attack of la grippe in 1894. Has dry hacking cough, with gelatinous expectoration, containing bronchial and alveolar epithelium in a state of fatty metamorphosis, streaked with blood. Temperature, 101 degrees. Loss of appetite and dyspeptic symptoms. Inspiration of cog-wheel character, expiration high-pitched and dullness on percussion. Patient has lost about 30 pounds within last few months. Weighed on January 2d, 145 pounds. Blood count, 45 per cent.; hemoglobin, 3,000,000 red cells, 7,500 white cells. Treatment began with one tablespoonful doses of liquid peptonoids with creosote every four hours. Patient slowly improved, and on June 16th doses were doubled to two tablespoonfuls every four hours. Hereafter a rapid improvement took place. July 1st, patient's cough has disappeared, no bacilli in sputum, appetite good, weight 151 pounds. This treatment was continued till July 26th, when patient left the hospital apparently well. Weight, 155 pounds; blood examination, hemoglobin 62 per cent., red cells 3,650,000, white cells 7,200; no cough, good appetite.

CASE II.—E. W., male, aged 20; family history, tubercular. Admitted June 9, 1898. Hacking cough, purulent expectoration, temperature 100 degrees, night sweats, loss of appetite and weight; blood examination, 43 per cent. hemoglobin, 2,700,000 red cells, 7,000 white cells; weight, 98 pounds; examination of sputum, bronchial and alveolar epithelium, bacilli. Same treatment as in Case I. began June 9th. Patient improved. June 26th, coughs but little, no bacilli in sputum, appetite good, weight 103 pounds. July 13th discharged, apparently well, no cough, no night sweats, appetite ravenous, weight 105 pounds; blood count, hemoglobin 61 per cent., red cells 3,600,000, white cells 6,800.

All tubercular cases under my observation improved under this treatment, while others under plain doses of creosote gtt v. to xx. showed but little improvement.

---

### ALBUMINURIA IN THE PUERPERIUM.

MABLEY, H.C., Cleveland, O., (*Cleveland Jour. of Med.*, March, 1899): Albuminuria occurring in the puerperium may be due to (*a*) a persistence of the renal conditions commonly present before and during labor, and known as the "kidney of pregnancy." Explaining these changes by the extra amount of work thrown upon the urinary organs during gestation, it is not difficult to account for a small quantity of albumin occurring in the urine during the period following labor, when such heavy demands are made upon the excretory functions. If we adopt the other, and perhaps more generally accepted theory, that the kidney of pregnancy with accompanying albuminuria has its cause in increased intraabdominal tension with consequent mechanic pressure upon the renal veins and ureters, we will look for a somewhat rapid diminution of the albumin following labor; and its continued presence in large or increasing quantity would lead us to seek for other factors in its causation.

(*b*) Albuminuria may also be looked for during the lying-in period in women suffering from a nephritis, which has either developed during pregnancy, or, existing prior to that time, in a chronic, or perhaps latent form, has been aggravated by the processes of gestation and parturition.

(*c*) Nephritis, depending upon an infection of the genital organs, is not uncommon in the puerperium, and here the amount of albumin seems often to vary directly with the extent of the lesions in the pelvis. Nephritis has been especially mentioned as a complication of uterine phlebitis and lymphangitis.

(*d*) Mammary inflammation may cause nephritis. Norris refers to a case which developed on the fourteenth day after labor in a patient suffering from a virulent abscess.

(*e*) It is conceivable also that a nephritis might occur in the puerperium, as an intercurrent affection, resulting from cold or toxic agents. At this time, moreover, the conditions present in the kidneys, while not of themselves amounting to disease, might tend to render these organs particularly susceptible to influences of such a nature.

(*f*) Albuminuria in women recently confined may be due also to catarrhal or suppurative affections of the bladder and ureters, to pyelitis, and in rare instances to perinephritic abscess.

(*g*) Finally, valvular lesions of the heart, with a failure of compensation preceding or following labor, constitute a possible cause for the appearance of albumin and casts in the urine at this time.

---

RUPTURE OF THE DRUM-HEAD NOT NECESSARILY INCURABLE.

LAUTENBACH, LOUIS J., of Philadelphia (Abstract *St. Louis Clinique*), says: In considering the question of the healing of perforations, two main propositions must engage our attention :

1. We must be sure that the structures within the perforated drum-head are not in a state of inflammation. If there be any disease present this must be thoroughly conquered before any serious effort is made to heal the membrane. When this inflammation is overcome, then we commence to heal the perforations, and under ordinary conditions the closure is easily accomplished.

2. If after the inflammation has subsided healing does not occur promptly, it is probably due to cicatrisation of the edges of the broken drum-head, and means must be adopted to renew these edges by making them more active and ready for healing.

By careful attention to these requisites, of curing the disease within the membrane, and of renewing and freshening the edges of the perforation whenever there seems a deficiency of reparative power, we will succeed in curing almost all cases of perforation. We will fail in few cases unless the perforations be of tubercular origin.

Burnett says: "The popular impression that the membrana tympani, once perforated, can never be healed, is a wrong one. The drum-head on the contrary, has great power of healing and restoration, as shown by Dr. H. N. Spencer and others. A simple slit in it will heal in a few hours if there be no inflammation in the drum cavity. Larger and even gaping perforations, caused by disease, tend to heal, unless the disease in the tympanum keeps up and leads to a cicatrisation of the edges of the opening in the membrana tympani."

Many years ago I treated a young girl, Mary H., who a few days before had the drum-head broken by efforts in removing a stone from the ear, and although several days had elapsed since the injury, and the drum-head and the surrounding tissues were considerably inflamed, there being no healing apparent, yet within a few weeks the drum-head was perfectly healed, and the hearing was practically perfect. Examining her ears several years later, I found the drum-head perfect in every respect, and the hearing on the injured side extraordinarily good, it being remarkably better than the average hearing, as well as better than the uninjured ear.

---

### SLEEP IN THE TREATMENT OF DISEASE.

Evart, Wm., in an address delivered before the Harveian Society (*British Medical Journal*, December 17, 1898,) on Disease and its Treatment, has the following to say on the value of sleep:

"Sleep has two offices, both fulfilled in the long sleep of the night, which it is our usual endeavor to secure for our patients—namely, that of favoring the slow changes of repair, and that of interrupting consciousness by uncoupling the chain of neurons, or by relaxing protoplasmic tension or tone. This relief of tension is, it would seem, the only office performed by the shorter spells of sleep, and therefore the two forms of sleep suggest two therapeutic objects. The night's sleep which comes without any drugs may need to be bettered, and in improving the quality of spontaneous sleep our help is often of value. It might also need to be prolonged.

"The systematic prolongation of sleep for the cure of disease is one of our opportunities hitherto little used. In chorea, sleep entirely subdues the muscular agitation, and this observation has led to the attempt to arrest the disease by prolonging sleep for considerable periods. A complication arises in connection with alimentation which in this disease, as in most other nervous troubles, is of primary importance. Partly for this reason, and because more than rest may be needed for a cure, the results hitherto reported have not sufficiently recommended the method.

"Prolonged narcosis has also been suggested in excessive wear and tear of the nervous system; and in various nervous affections, including the mental, its renewed trial, combined with suitable methods of feeding, might lead to encouraging results.

"Best suited, perhaps, to our every-day needs is a systematic resort to the shorter sleep. Like the light installments of food which restore the lost function of appetite and digestion, short sleep in the day may be essential to the cure of nocturnal insomnia. Our growing wealth in hypnotics warrants a hope that a suitable agent may yet be found which in that direction would minister to the health of the invalid, and might command the luxury of sleep at any opportune time for the convenience of the worker.

"Body rest as a systematic therapeutic agent has long found its place in our treatment for patients whom weakness alone, in the absence of medical advice, would not have compelled to take to their bed. To that class belong the frail women in whom the debility of anemia, of dyspepsia, and of overfatigue develop symptoms often mistaken for hysteria. Rest in bed is their first need. In the treatment of chlorosis this is now recognised as the essential element for a rapid recovery. Its methodical employment forms an essential part of the Weir-Mitchell plan. But its most striking instance is that of the open-air rest cure for phthisis, which within quite recent years has largely replaced at foreign sanatoria the previous method by muscular exercise."—*Medical Age.*

---

### DIFFERENTIAL DIAGNOSIS OF HEADACHES.

THE above is the title of a paper read by Joseph Mullen, M. D., before the Houston District Medical Association, and published in the *Memphis Lancet* for November. It deals with various forms of headaches and their causes and treatment, but the most striking paragraph is the following :

"Roughly speaking, there are about four pharyngeal conditions capable of producing headaches of such intensity as to draw the patient's and doctor's attention from the cause to the effect. They are adenoids, post-nasal catarrh, follicular pharyngitis, and enlarged tonsils. With the first and last you are very familiar—that is, adenoids and hypertrophied tonsils. The mouth-breathing, listless and dull facial expression so characteristic of their presence, so uniform are these, that they have become pathognomonic. The treatment calls for their removal. The headaches are usually dull, rarely sharp or excruciating, and are felt in any portion of the head. Like post-nasal catarrh and post-nasal enlargement of the turbinates, they may produce vertex head pain."

---

### THE HYPODERMIC USE OF MERCURY IN SYPHILIS.

JONES, S. S. (*Jour. of Cutaneous and Genito-Urinary Diseases*, December 2, 1898,) presents a few practical observations based on personal experience in private practice, and gives the result of his endeavor to rid the method of its most objectionable feature—namely, its tendency to occasion pain, inconvenience, and even great suffering. He had been inclined to look unfavorably upon the method from considerations of pain and annoyance, until he came to face the necessity for its use in a critical case of brain syphilis, which led him to seek some method for lessening its disadvantages.

For convenience in use, the author had a tablet made containing bichloride and sodium chloride, of each one-fourth grain, cocaine one-sixteenth grain, which he found could be readily serviceable and at hand.

The author believes the method superior to others : (1) Where danger threatens organs whose functions are vital or important, as lesions of brain, heart, eye or ear; (2) in ulcerations resembling tubercular or malignant disease, especially of the tongue; (3) in those cases where other methods are not tolerated or when time is valuable.

The injection should be made into muscle, the needle being washed after discharging syringe. The author, however, has found it not always possible to inject into muscle, and has found no ill-results from injection into the subcutaneous cellular tissue. He believes the most eligible sites to be in the cellular or muscular tissue between the scapulæ and the iliac crests.

---

THE RELATION OF PUBLIC PARKS TO PUBLIC HEALTH.—The relation of public parks to public health may not admit of mathematical demonstration, yet an intimate and vital relation does exist. Park literature and health-board reports contain many tables and illustrations of the importance of parks in cities; the efficacy of open air spaces and playgrounds; the benefit of trees and shrubs in localities where miasmatic and other diseases prevailed; of lives saved by pure air and sunshine—especially of children, who are more susceptible; of the conservation of vital and effective physical force, and of other hygienic and economic services rendered by public parks. The history of sanitation reveals wonderful transformations of slums to healthy homes, plagues stayed and death averted by the investigation of causes, intelligent expenditure of labor to overcome them and proper observance of the rules of health.—*Orlando B. Douglas.*

---

ECZEMA AND WASHING OF THE HANDS.

UNNA (*Mons. f. pr. Derm.*) explains that the aggravation following the use of water in eczema of the hands is not due to the action of water upon the eczematous surface, but to the drying up of the epidermis, with all its consequences—rhagades, if the washed eczematous hand is not protected from exposure to the air.—*Journal Cutaneous and Genito-Urinary Diseases.*

